# Calcified Peyronie’s Disease Frequency on Computed Tomography

**DOI:** 10.5152/tud.2022.21346

**Published:** 2022-05-01

**Authors:** Elif Gündoğdu, Emre Emekli

**Affiliations:** 1 Department of Radiology, Eskişehir Osmangazi University, Faculty of Medicine, Eskişehir, Turkey; 2 Department of Radiology, Etimesgut Şehit Sait Ertürk State Hospital, Ankara, Turkey

**Keywords:** Calcified peyronie disease, penis, computed tomography

## Abstract

**Objective:**

In computed tomography examinations performed for various reasons, calcified Peyronie’s disease can be incidentally detected. In this study, we aimed to evaluate the frequency of calcified Peyronie’s disease incidentally detected in patients with abdominal computed tomography.

**Material and methods:**

The images of male patients undergoing abdominal computed tomography between January 2019 and January 2020 were retrospectively evaluated for the presence of calcified Peyronie’s disease. 1968 patients remained after subtracting computed tomography scans for insufficient evaluation of the penis, evaluated for the presence of calcified Peyronie’s disease by two radiologists based on consensus. The localization, side, and the number of plaques were recorded.

**Results:**

: The computed tomography examination of 1968 patients revealed calcified Peyronie’s disease in 130 (6.6%) patients. Peyronie’s disease was bilateral in 73 patients (56.1%), and unilateral in 57 (43.9%). A single plaque was observed in 44 (33.9%) patients, and multiple plaques in 86 (66.1%). The plaques were located in the middle portion of the penis in 98, proximal penis in 92, and distal penis in 31 cases.

**Conclusion:**

Calcified Peyronie’s disease is incidentally detected on computed tomography examinations at a rate not rare. Peyronie’s disease tends to be multiple, bilateral, and localized in the middle portion of the penis.

**Main Points**

- Calcified Peyronie’s disease are incidentally detected in computed tomography examinations at a rate not rare.
- Calcified Peyronie’s disease are most frequently observed in the sixth and seventh decades of age.
- Peyronie’s disease tend to be multiple, bilateral, and localized in the middle portion of the penis.

## Introduction

Peyronie’s disease (PD) is a benign condition characterized by the formation of fibrous plaque in the tunica albuginea layer of the penis.^1,2^ It is an important condition that can cause painful penile erection, curvature deformity in the penis, shortened penile length, erectile dysfunction, and associated psychological problems.^3,4^ The prevalence of PD varies between 0.4% and 23% in different case series.^5-7^ An increase in prevalence is observed with age.^7^

Despite many hypotheses, the true etiopathogenesis of PD remains unclear.^3,8-10^ The most accepted theory is inflammation and fibrin deposition that occur as a result of the exposure of the tunica albuginea to repeated microtraumas.^3,8-10^ Although the etiopathogenesis of PD is not precisely known, it is considered to be multifactorial.^11^ It has been shown to be associated with genital-perineal iatrogenic or non-iatrogenic trauma, hypogonadism, smoking, obesity, diabetes, hypertension, collagen tissue disease, Dupuytren’s contracture, and metabolic syndrome.^12^

The diagnosis of PD is usually made clinically based on patient history and physical examination findings.^13^ Imaging is required to evaluate the location and size of plaques, whether they are calcified, and the relationship between plaque and penile vascular structures.^11^ However, it is believed that patients are reluctant to undergo an evaluation for diagnosis and treatment options.^14^ Therefore, it is generally considered that the true prevalence of PD is higher than reported. This study aimed to investigate the frequency of incidentally detected calcified PD in patients undergoing abdominal CT for other reasons.

## Material and Methods

Before the study, the approval of the local ethics committee was obtained (date: March 17, 2020 number: 25403353-050.99-E.39371). The study was conducted in accordance with the principles of the Declaration of Helsinki. The study was conducted in a tertiary-care hospital. All image data used in this study were obtained from routine imaging at our hospital. Datasets were evaluated retrospectively. The study is a cross-sectional study and STROBE guidelines were followed in it.

### Study Prticipants

Medical data of patients including patient medical history, co-morbid conditions, medications and complaints were obtained from the hospital information system. Computed tomography images of patients obtained from hospital picture archiving and communication system.

**Figure 1.**
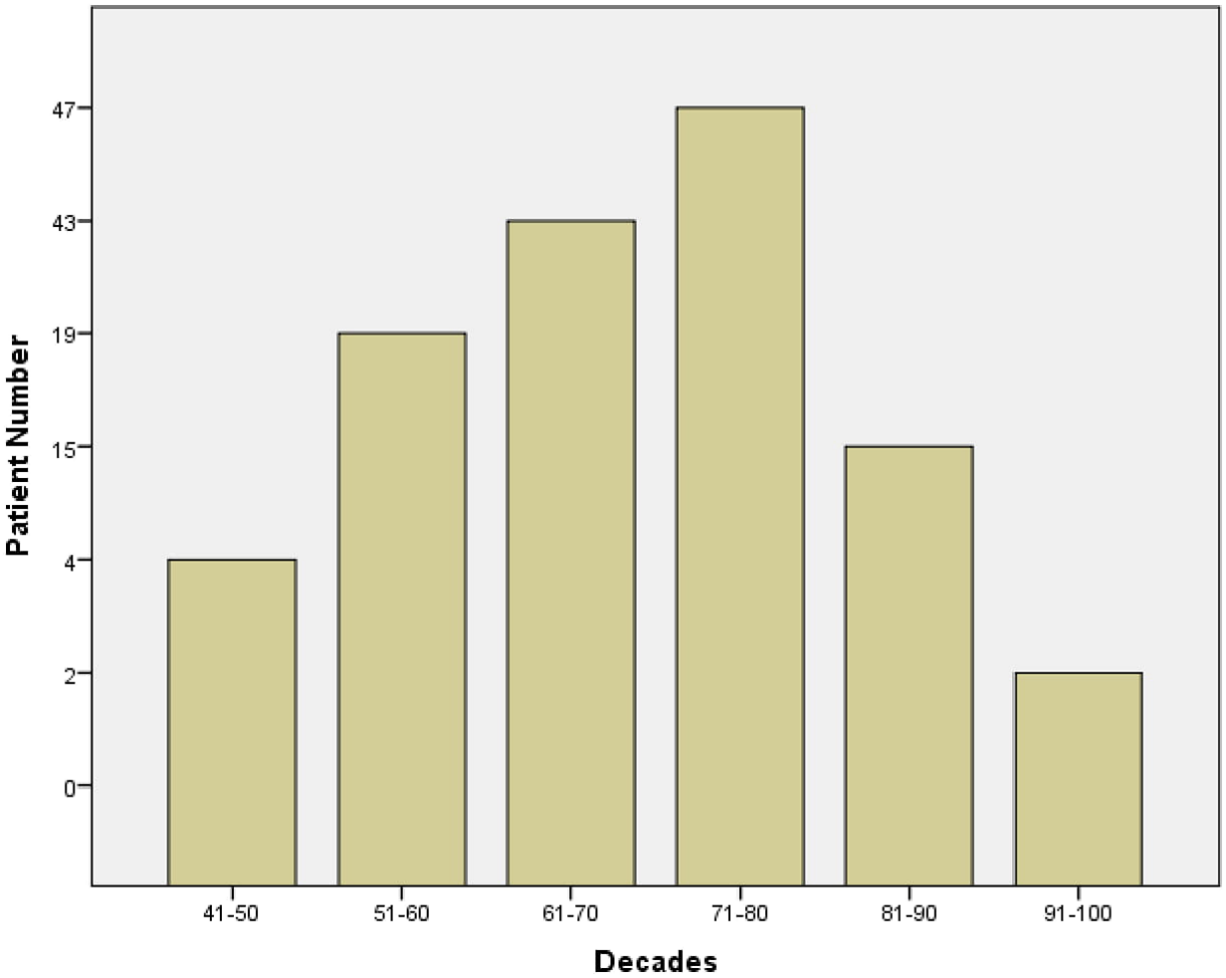
The number of calcified Peyronie’s plaques according to patient age by a decade.

The images of male patients that underwent an abdominal CT examination for various reasons (malignancy screening, tumor staging, abdominal pain etiology, urinary stone disease, unexplained fever, weight loss, ileus and abdominal aort aneurysm) between January 2019 and January 2020 were retrospectively evaluated. Computed tomography examinations of 2845 patients who applied from outpatient clinics other than emergency departmant were evaluated in terms of examination suitability and whether the entire penis was included in the examination. Eight hundred seventy-seven patients with inadequate scans (patient-induced motion artifacts, metallic prosthesis that created artifacts in the pelvic region, or cases where the skin surface was not completely covered, patients in whom the entire penis is not included in the examination) were excluded from the study. The CT scans of the remaining 1968 patients were evaluated for the presence of calcified PD. The CT images were evaluated by two radiologists based on consensus. The normal corpora cavernosa appears on CT as two adjoining rounded structures of homogeneous density surrounded by a thin and hyperdense border corresponding to the tunica albuginea^15^ (Figure 1). Tunica albuginea has a uniform thickness and is always well-distinguishable from the surrounding tissues.^15^ Cavernosal artery calcifications are located in the central part of the corpus cavernosum, while calcified PD are located on the tunica albuginea at the periphery. The calcified PD are seen markedly hyperdense area on the thin, lineer hyperdence tunica albuginea on CT. Multi-plane images were used for the detection of plaques on CT and we evaluated the penis for the presence of calcified PD as mentioned above.

In patients with plaques, the localization of the plaques (proximal, middle, and distal penis) and their side (right, left, and bilateral) were recorded. Plaque localization was determined by dividing the flaccid penis into three equal compartments.

### Computed Tomography Protocols

Computed tomography imaging was performed using 64-slice (Toshiba, Aquillon 64, Japan) or 128-slice (GE, Revolution EVO, USA) multi-detector CT scanners. The subjects were examined in a supine position with their arms extended above their heads. The CT parameters were as follows: 1:1 pitch, 200-250 mAs, 120 kVp, and 0.5-0.625 isotropic spatial resolution.

### Statistical Analysis

SPSS software v.22 (IBM SPSS Corp.; Armonk, NY, USA) was used for statistical analysis. Descriptive statistics were presented as mean, standard deviation, median, minimum and maximum values for the continuous data and percentage values for the discrete data.

## Results

In the CT examination of 1968 male patients, 130 (6.60%) cases were found to have calcified PD. The ages of the patients with PD varied between 47 and 91 years (mean age 69.50 ± 9.85 years). The number of patients according to each decade of age is shown in Figure 1. The number of patients and percentages by age group are given in Table 1.

None of the 130 patients had penile complaints about CT ­indication. In the medical records, it was not possible to find out whether detailed inquiries were made on penile complaints. The comorbid diseases of the patients were as follows: 39 diabetes mellitus, 91 hypertension, 66 hyperlipidemia, 52 atherosclerotic heart disease, 31 congestive heart failure, 14 liver failure, 21 kidney failure, 9 solid organ malignancy. And 21 patients were using oral antidiabetic, 20 patients were using insulin, 88 patients were using antihypertensive, and 7 patients were using various antineoplastic chemotherapeutics due to malignancy.

**Figure 2.**
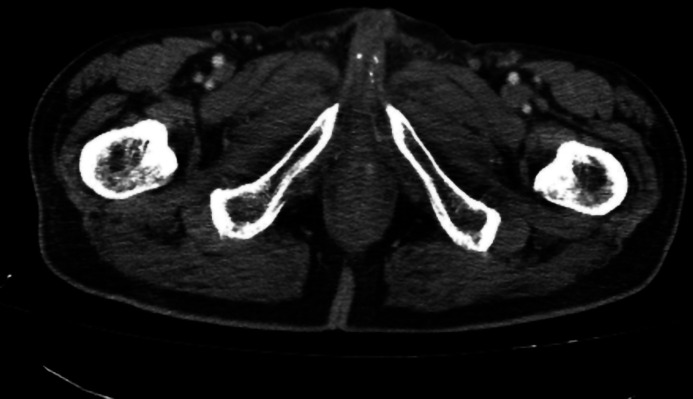
Computed tomography images show calcified Peyronie's plaques on both sides of the penis, multiple on the left-sided.

**Figure 3.**
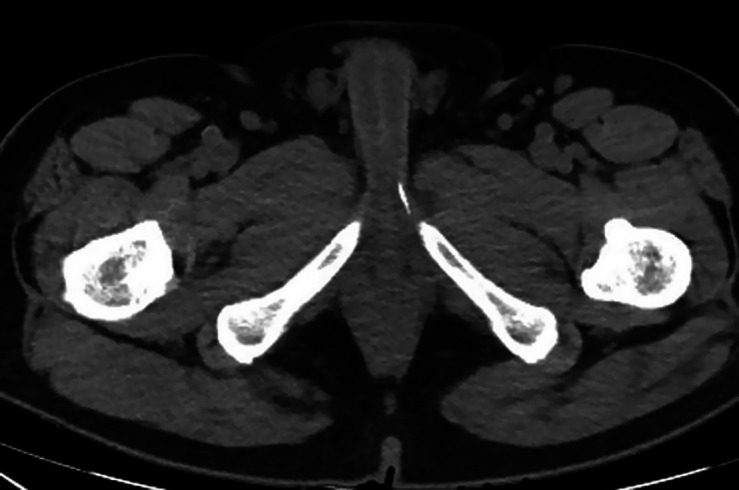
Computed tomography images showing single calcified Peyronie's plaques on left proximal sides of the penis.

A plaque was detected on both the right and left sides of the penis in 73 (56.15%) of 130 patients, while unilateral calcified plaque was detected in 57 (43.85%) patients. While 44 (33.85%) patients had a single plaque, 86 (66.15%) patients had more than one plaque (Figure 2). The plaques were located in the middle portion of the penis in 98 patients, proximal penis in 92 patients, and distal penis in 31 patients (Figure 3). Table 2 presents the data on the side, number and localization of plaques.

## Discussion

In this study, the frequency of calcified PD incidentally detected on CT was found to be 6.60%. It was observed that the majority of the plaques (69.23%) were detected in individuals aged between 61 and 80 years. The plaque incidence increases with decades. However, the number of patients over the age of 80 was less, so the number of patients with plaque showed an accumulation in the 61-80 age group. The prevalence of bilateral PD was more common than that of unilateral PD. In addition, more than one plaque was observed in 66.15% of the patients. While most of the plaques were seen in the middle portion of the penis, the least common localization was the distal penis.

In the literature, the global prevalence of PD is observed to have a wide range of 0.4-23%.^5,6^ The reason for this wide range is due to the different methodologies used in the diagnosis of PD, the patient being symptomatic or asymptomatic, and the varying characteristics of the patient populations. There are some personal risk factors with a well-defined relationship with PD, such as smoking, diabetes, history of urogenital surgery, and presence of Dupuytren’s contracture.^11,12^ It is expected that the prevalence of PD differs in studies conducted with the general population and patient groups with these risk factors. The individuals in our study group were from a general patient population. Our prevalence (6.60%) was in the range reported in the literature. However, to date, studies conducted have mostly used physical examination findings in the diagnosis of PD. In contrast, we evaluated the prevalence of calcified PD using CT. It is reported that calcified plaques constitute approximately 25-35% of all PD.^16^ Computed tomography is an excellent imaging method for the detection of calcified plaques, but it is known that soft-fibrotic plaques are not well visualized.^17^ For this reason, it is possible that there were two or three times more fibrotic plaques than our rate of PD, which we were not able to detect on CT in our study population.

Most of the plaques detected in our study (96.92%) were observed in individuals over 50 years old, and were observed most frequently in the seventh decade, followed by the sixth decade of age. The prevelance of plaque increases with the decade. Segunda et al^18^ reported that the frequency of plaques was higher in individuals over 50 years old. In that study, unlike ours, the rate of PD was greater among the patients in the fourth decade. This is because they also evaluated fibrous plaques. In this age group, fibrous plaques may be more common than calcified plaques. In another study, Habous et al^8^ reported the highest incidence of PD in the seventh decade at the (30%), which is similar to our rate of 36%. However, Habous et al^8^ noted that the incidence of PD was higher in the 20-50 age group (37.2%) while this rate was only 3% in our study. We consider that the reason for this different finding is the absence of a fibrous plaque evaluation in our work.

In this study, we found that 56.15% of calcified plaques were in the tunica albuginea of both corpus cavernosa, and 66.1% of the patients had more than one plaque. There is no clear detailed information on this subject in the literature. Since a physical examination is often used together with the patient’s history in the diagnosis of PD, it is not possible to clearly verify the number and distribution of plaques. In addition, septal plaques and punctate scars are non-palpable, and thus it is possible to overlook them on physical examination.^13^ Therefore, objective imaging methods are required. To this end, color Doppler ultrasonography is often used; however, it is also reported that the presence of incidentally detected plaques is not uncommon in CT.^19^ The results of our study are important in terms of showing the frequency of incidentally detected calcified PD and the prevalence of plaques on CT. According to the results of our study, the presence of multiple plaques was approximately two times more frequent than the rate of a single plaque. Therefore, when a plaque is detected by both physical examination and other imaging methods, clinicians should carefully consider the possibility of the presence of more plaques.

In our study, we determined that calcified PD were most common in the middle portion of the penis, mostly more than one compartment was affected, and the least frequent location was the distal penis. Some authors reported the most common location as the proximal and middle penis, and the least location as the distal penis.^20^ Our results are consistent with the data in the literature. Segunda et al showed that the most common localization of plaques was the distal ¼ part of the penis. In that study, the authors made a diagnosis based on physical examination findings and also evaluated fibrous plates. In addition, the fact that the penis was examined in four parts in this study may have affected the results. Since our study was carried out using an imaging method, it provided a more objective evaluation compared to previous studies. In the literature, Arafa et al.^21^ who used ultrasonography, found the most common plaque localization as the middle portion of the penis, which is in agreement with the findings we obtained from our imaging study.

One of the most important limitations of our study is its retrospective nature. So, this we cannot correlate the presence of calcified plaques with clinical symptoms such as penile curvature or palpable plaque because of in adequate data about these in medical records. In addition, evaluation of only calcified plaques may be considered as a limitation. However, our research being carried out with a large population and using an imaging method (CT) that allows an objective evaluation of PD diagnosis are among the strengths of our study. In addition, being a large-scale study providing data on the localization, number, and side of plaques which is a relatively less researched area in the literature, the results presented can guide clinicians in terms of the treatment decision. It is estimated that the incidence of PP is higher than reported due to patients feeling embarrassed and being reluctant to seek professional help. Therefore, similar to the methodology used in the current work, further cross-sectional screening studies will be valuable in evaluating the true prevalence of PD.

In conclusion, calcified PD is incidentally detected in CT examinations at a rate that cannot be considered rare. Calcified PD is most frequently observed in the sixth and seventh decades of age. They tend to be multiple in number, localized bilaterally, and in the middle portion of the penis.

## Figures and Tables

**Table 1.** The Number of Patients and Percentages by Decades

Decades	Number of Patients with PD (n)	Number of Patients (n)	Prevelance (Total) (%)	Prevelance (Decades) (%)
41-50	4	311	0.20	1.28
51-60	19	368	0.96	5.16
61-70	43	575	2.18	7.47
71-80	47	557	2.38	8.43
81-90	15	139	0.76	10.79
91-100	2	18	0.10	11.11
**Total**	130	1968	6.60	

**Table 2.** The Characteristics and Frequency of the Detected Plaques

Plaques	Number (n)	Percentile (%)
*Side*	130	
Bilateral	73	56.15
Right	31	23.85
Left	26	20
*Number*	130	
Single	44	33.84
Multiple	86	66.16
*Localization*	130	
Isolated proximal	29	22.30
Isolated middle	32	24.61
Isolated distal	3	2.30
Proximal and middle	38	29.24
Middle and distal	3	2.30
Proximal, middle and distal	25	19.24

## References

[1] Ralph D Gonzalez-Cadavid N Mirone V et al. The management of Peyronie’s disease: evidence-based 2010 guidelines. J Sex Med. 2010;7(7):2359 2374. 10.1111/j.1743-6109.2010.01850.x) 20497306

[2] Ten Dam EPM van Driel MF de Jong IJ Werker PMN Bank RA . Glimpses into the molecular pathogenesis of Peyronie’s disease. Aging Male. 2020;23(5):962 970. 10.1080/13685538.2019.1643311) 31335242

[3] Garaffa G Trost LW Serefoglu EC Ralph D Hellstrom WJ . Understanding the course of Peyronie’s disease. Int J Clin Pract. 2013;67(8):781 788. 10.1111/ijcp.12129) 23869679

[4] Gunes M Aslan R Eryılmaz R Demir H Taken K . Levels of serum trace elements in patients with Peyronie. Aging Male. 2018;17:1 4. 10.1080/13685538.2018.1474195 29768978

[5] Lindsay MB Schain DM Grambsch P Benson RC Beard CM Kurland LT . The incidence of Peyronie’s disease in Rochester, Minnesota, 1950 through 1984. J Urol. 1991;146(4):1007 1009. 10.1016/s0022-5347(17)37988-0) 1895413

[6] Tefekli A Kandirali E Erol B Tunc M Kadioglu A . Peyronie’s disease: a silent consequence of diabetes mellitus. Asian J Androl. 2006;8(1):75 79. 10.1111/j.1745-7262.2006.00099.x) 16372122

[7] Kadioglu A Dincer M Salabas E Culha MG Akdere H Cilesiz NC . A population-based study of Peyronie’s disease in turkey: prevalence and related comorbidities. Sex Med. 2020;8(4):679 685. 10.1016/j.esxm.2020.09.002) 33243422 PMC7691981

[8] Habous M Malkawi I Han E et al. Peyronie’s disease is common in poorly controlled diabetics but is not associated with the Metabolic Syndrome. Urol Ann. 2019;11(3):252 256. 10.4103/UA.UA_164_18) 31413501 PMC6676820

[9] Özbir S Değirmentepe RB Atalay HA et al. The role of inflammatory parameters (neutrophil-to-lymphocyte ratio, platelet-to-lymphocyte ratio, and monocyte-to-eosinophil ratio) in patients with Peyronie’s disease. Andrology. 2020;8(2):348 352. 10.1111/andr.12702) 31512411

[10] De Rose AF Mantica G Bocca B Szpytko A Van der Merwe A Terrone C . Supporting the role of penile trauma and micro-trauma in the etiology of Peyronie’s disease. Prospective observational study using the electronic microscope to examine two types of plaques. Aging Male. 2019;16:1 6. 10.1080/13685538.2019.1586870 30879382

[11] Bilgutay AN Pastuszak AW . Peyronie’s disease: a review of etiology, diagnosis, and management. Curr Sex Health Rep. 2015;7(2):117 131. 10.1007/s11930-015-0045-y) 26279643 PMC4535719

[12] Bjekic MD Vlajinac HD Sipetic SB Marinkovic JM Marinkovic JM . Risk factors for Peyronie’s disease: a case-control study. BJU Int. 2006;97(3):570 574. 10.1111/j.1464-410X.2006.05969.x) 16469028

[13] McCauley JF Dean RC . Diagnostic utility of penile ultrasound in Peyronie’s disease. World J Urol. 2020;38(2):263 268. 10.1007/s00345-019-02928-y) 31606787

[14] Shindel AW Sweet G Thieu W Durbin-Johnson B Rothschild J Szabo R . Prevalence of Peyronie’s disease-like symptoms in men presenting With Dupuytren contractures. Sex Med. 2017;5(3):e135 e141. 10.1016/j.esxm.2017.06.001) 28676223 PMC5562496

[15] Rollandi GA Tentarelli T Vespier M . Computed tomographic findings in Peyronie’s disease. Urol Radiol. 1985;7(3):153 156. 10.1007/BF02926875) 4071858

[16] Wymer K Ziegelmann M Savage J Kohler T Trost L . Plaque calcification: an important predictor of collagenase Clostridium histolyticum treatment outcomes for men with Peyronie’s disease. Urology. 2018;119:109 114. 10.1016/j.urology.2018.06.003) 29908867

[17] Parmar M Masterson JM Masterson TA . The role of imaging in the diagnosis and management of Peyronie’s disease. Curr Opin Urol. 2020;30(3):283 289. 10.1097/MOU.0000000000000754) 32205808

[18] Segundo A Glina S . Prevalence, risk factors, and erectile dysfunction associated with Peyronie’s disease among men seeking urological care. Sex Med. 2020;8(2):230 236. 10.1016/j.esxm.2019.11.002) 32007472 PMC7261680

[19] Shenoy-Bhangle A Perez-Johnston R Singh A . Penile imaging. Radiol Clin North Am. 2012;50(6):1167 1181. 10.1016/j.rcl.2012.08.009) 23122044

[20] Kalokairinou K Konstantinidis C Domazou M Kalogeropoulos T Kosmidis P Gekas A . US imaging in Peyronie’s disease. J Clin Imaging Sci. 2012;2:63. 10.4103/2156-7514.103053) PMC3515929 23230545

[21] Arafa M Eid H El-Badry A Ezz-Eldine K Shamloul R . The prevalence of Peyronie’s disease in diabetic patients with erectile dysfunction. Int J Impot Res. 2007;19(2):213 217. 10.1038/sj.ijir.3901518) 16915304

